# Congenital Disorders of Glycosylation from a Neurological Perspective

**DOI:** 10.3390/brainsci11010088

**Published:** 2021-01-11

**Authors:** Justyna Paprocka, Aleksandra Jezela-Stanek, Anna Tylki-Szymańska, Stephanie Grunewald

**Affiliations:** 1 Department of Pediatric Neurology, Faculty of Medical Science in Katowice, Medical University of Silesia, 40-752 Katowice, Poland; 2 Department of Genetics and Clinical Immunology, National Institute of Tuberculosis and Lung Diseases, 01-138 Warsaw, Poland; jezela@gmail.com; 3 Department of Pediatrics, Nutrition and Metabolic Diseases, The Children’s Memorial Health Institute, W 04-730 Warsaw, Poland; atylki@op.pl; 4 NIHR Biomedical Research Center (BRC), Metabolic Unit, Great Ormond Street Hospital and Institute of Child Health, University College London, London SE1 9RT, UK; Stephanie.Grunewald@gosh.nhs.uk

**Keywords:** congenital disorders of glycosylation, neurological symptoms, epilepsy, neuroimaging-stroke-like episodes, ataxia, autistic spectrum disorders, CDG-related genes

## Abstract

Most plasma proteins, cell membrane proteins and other proteins are glycoproteins with sugar chains attached to the polypeptide-glycans. Glycosylation is the main element of the post-translational transformation of most human proteins. Since glycosylation processes are necessary for many different biological processes, patients present a diverse spectrum of phenotypes and severity of symptoms. The most frequently observed neurological symptoms in congenital disorders of glycosylation (CDG) are: epilepsy, intellectual disability, myopathies, neuropathies and stroke-like episodes. Epilepsy is seen in many CDG subtypes and particularly present in the case of mutations in the following genes: *ALG13*, *DOLK*, *DPAGT1*, *SLC35A2*, *ST3GAL3*, *PIGA*, *PIGW*, *ST3GAL5*. On brain neuroimaging, atrophic changes of the cerebellum and cerebrum are frequently seen. Brain malformations particularly in the group of dystroglycanopathies are reported. Despite the growing number of CDG patients in the world and often neurological symptoms dominating in the clinical picture, the number of performed screening tests eg transferrin isoforms is systematically decreasing as broadened genetic testing is recently more favored. The aim of the review is the summary of selected neurological symptoms in CDG described in the literature in one paper. It is especially important for pediatric neurologists not experienced in the field of metabolic medicine. It may help to facilitate the diagnosis of this expanding group of disorders. Biochemically, this paper focuses on protein glycosylation abnormalities.

## 1. Introduction

Glycosylation is the most common post-translational modification of proteins and lipids and its biological role is crucial in the processes of development, growth and functioning of the body. Most of our body’s proteins are essentially glycoproteins composed of a polypeptide and oligosaccharide glycans. The glycans are attached to asparagine residues by an N-glycosidic bond or to serines or threonines by an O-glycosidic linkage. N-glycans are mainly composed of N-acetylglucosamine (GlcNAc), mannose (Man), galactose (Gal), sialic acid (NeuNAc) and fucose (Fuc). O-glycans (of the mucin-type) are sugar chains less branched than N-glycans and are usually bi-antennic [1,2,3]. They are made up of N-acetylgalactosamine (GalNAc), N-acetylglucosamine, galactose, fucose and sialic acid [1,2,3]. Their synthesis involves several stages located in the cytoplasm, endoplasmic reticulum (ER) and Golgi apparatus. Incorrect glycosylation of proteins can disrupt the functions of many organs and tissues, underlying complex clinical symptomatology and organ pathology. N-and O-glycosylation deficiency is characterized by a group of genetically conditioned metabolic defects called congenital glycosylation disorders. N-and O-glycans are commonly attached to proteins of all tissues and organs. They usually occupy the surface of protein molecules (the weight of the glycan can even exceed the weight of the protein) and are tissue and developmental specific. The biological role of N-and O-glycans are diverse and include contributing to maintain the structure and integrity of tissues, protecting glycoproteins against proteolysis, enabling correct folding of the polypeptide in ER (N-glycans), modulating the activity of hormones, receptors, growth factors, enzymes and other “active” glycoproteins, enabling the correct location of glycoproteins within and outside the cell, taking part in recognition of the “cell to cell” and the cell to substance basic, serving as a protective barrier for tissue against viruses and bacteria [1,2,3].

The basic test in the diagnosis of N-glycosylation disorders is the determination of transferrin isoforms. Using the method of isoelectric focusing (IEF), an abnormal cathodic shift of the isoforms means a defect in the biosynthesis of the glycans and is a biochemical marker of congenital disorders of glycosylation (CDG). Protein glycosylation disorders can be divided into the following groups [1,2,3]:

- disorders of N-glycosylation
- disorders of O-glycosylation
- disorders of glycosylphosphatidylinositol (GPI) anchor and glycolipid anchor
- various disorders of glycosylation pathways (disorders of O-mannosylation, follicular transport)

The most frequently diagnosed group are N-glycosylation disorders. Depending on the severity, this might be mild to severe phenotypes and from single-to multi-organ symptoms [4].

Historically, the classification of congenital disorders of N-glycosylation was as follows [1,2,3]:

Type I—included the subtypes with defects in the assembly of a primary lipid-bound oligosaccharide (LLO) and its co-translational transfer to the polypeptide chain, located in the cytosol and endoplasmic reticulum (ER). Defects caused incomplete additions of N-glycosylation sites on protein molecules.

Type II—included glycosylation defects in the processing and remodeling of N-glycans located in the ER and Golgi apparatus

Evaluation of transferrin isoforms is still the method of choice in diagnostics of CDG abnormalities, though it has limitations. More than 140 different CDG types associated with abnormal protein glycosylation are currently described [1,2]. N-glycosylation defects are responsible for more than 70 types [1,2]. The next diagnostic stage is based on the assessment of biochemical, enzyme and genetic testing, either by single gene, CDG panel or WES/WGS testing. The early and specific diagnosis of the CDG subtype is essential because of the possibility of pharmacological treatment of selected types of CDG such as galactose in case of PGM1-CDG and SLC35A2-CDG, fucose in SLC35C1-CDG, Mn^2+^ in TMEM165-CDG, mannose in MPI-CDG or transplantation of liver in MPI-CDG or heart in DOLK-CDG [1,2,3,4,5]. PMM2-CDG is the most common type in Europe, followed by ALG6-CDG and SRD5A3-CDG. PMM2-CDG and ALG6-CDG are associated with the longest survival [1,2,3,4,5].

Although CDG is a multisystemic disease, the symptoms focus on neurological abnormalities. Neurological symptoms include psychomotor retardation/cognitive disorders, epileptic seizures (ESe), hypotonia, ataxia, polyneuropathy, stroke-like events [6]. The implications of disordered glycosylation in neural development may be relevant for the development and maintenance of normal cognitive functions. For example, in the most common type of CDG–PMM-CDG detailed percentage incidence of particular neurological symptoms was determined as follows [7]:

- psychomotor retardation/intellectual disability (ID) (90–96%)
- ataxia/cerebellar syndrome (96%)
- cerebellar atrophy (95%)
- hypotonia with frequent hyporeflexia (92%)
- strabismus (84%)
- ESe (11–12%), abnormal EEG findings (69%)
- peripheral neuropathy (53%)
- retinitis pigmentosa (22%)
- nystagmus (9.5%)
- thrombotic episodes: 89.3%-patients under ten years of age, 68% at least one stroke incident (provoking factors: fever, head injury)

## 2. Epileptic Encephalopathies

The etiology of epilepsy in CDG remains unclear and is supposed to be multifactorial as many genetic variants are responsible for their occurrence. The term “epileptic encephalopathy” was redefined in the Berg et al. report as to where the epileptic activity itself contributes to severe cognitive and behavioral impairments above and beyond what might be expected from the underlying pathology alone [8]. In an epileptic encephalopathy, the abundant epileptiform activity interferes with development resulting in cognitive slowing and often regression and sometimes is associated with psychiatric and behavioral consequences [8,9]. Most children with epilepsy have complete seizure control with current anti-seizures medications (ASMs) and other available treatments but approximately 30% suffer from refractory epilepsy, despite recent therapeutic developments [10]. Along with the development and availability of genetic analysis using next-generation sequencing (NGS) in the last decade, increasing knowledge about the genes and gene families that can be involved in epileptogenesis and help gain insight into the pathomechanisms underlying the different forms of epilepsy. Next-generation sequencing methodology is a promising, cost-effective approach to personalized treatment applicable in clinical practice. However, there are still many challenges in methodology, specifically in the analysis of genes residing in complex genomic regions and in result interpretation, along with ethical issues that need to be faced with and resolved before the application of this methodology in pharmacogenomics.

Referring to early-onset epileptic encephalopathies reported by Fiumara et al. [11], ESe were present in 11% of genetically confirmed PMM2-CDG. Epilepsy may be recognized in most CDG subtypes. Patients with ESe and CDG can be diagnostically challenging given the limited number of patients described in detail (the type of seizures, epileptic syndrome, treatment). Based on literature data it seems that ESe in the majority of CDG patients can be treated with one or two ASMs and tend to disappear with age.

Hypotheses of ESe pathomechanisms in CDG comprise [1,2,3,4,5,6,11,12]:

- a disturbed balance between excitatory and inhibitory neuronal activity (improper function of the voltage-gated ion-channels proteins within the cell membrane connected with lack of N-glycans: improper folding, shifted gating),
- defective glycosylation of signal transducers such as receptors,
- in specific CDG subtypes: mutations in the X-linked UDP-Galactose transporter SLC35A2, in the UDP-GlcNAc transporter SLC35A3, decreased localized production of the protein-free GAG hyaluronan, deletions in glycoprotein neurexin1 (NRXN1),
- congenital structural brain abnormalities most frequently connected with neuronal migration disorders (disturbed O-mannosylation of dystroglycans, different proteins like cadherin, abolishing polysialic acid, which presents on N-glycans in NCAM),
- regulation of GABA_A_R function (ALG13-CDG)

The CDG types not connected with special epileptic syndromes are for example ALG6-CDG, DPM2-CDG [13,14].

For ALG6-CDG, more than 90% experience epilepsy, ataxia and proximal muscle weakness. Behavioral changes, autistic features, depressive symptoms are quite frequent [6,14].

Barone et al. described 3 patients with DPM2-CDG and refractory epilepsy (tonic seizures) with onset between 1 week and five months, retarded/absent psychomotor development, severe hypotonia with preserved deep tendon reflexes, microcephaly and contractures, elevated creatine kinase (CK) levels (together with increased transaminases) [13]. EEG showed bursts of multiple paroxysmal spikes. Brain MRI disclosed cerebral periventricular and subcortical white matter atrophy and in two patients additional cerebellar hypoplasia and severe vermis hypoplasia [14]. The clinical spectrum of DPM2-CDG may overlap with DPM1-CDG and probably with GPI-anchor formation.

### 2.1. Epileptic Spasms(ESp)/West Syndrome

Given the large number of CDG patients described before 2017, when the new International League Against Epilepsy (ILAE) classification of ESe appeared, it is currently not possible to present ESe in the course of CDG syndrome according to the new classification [10]. Due to difficulties with retrospective verification of seizure semiology, epileptic encephalopathies were presented as: ESp/West syndrome, Ohtahara syndrome, early myoclonic encephalopathy of infancy, epilepsy of infancy with migrating focal seizures. However, we should remember that for many well recognized epileptic syndromes, such as childhood absence epilepsy, West syndrome and Dravet syndrome there has never been a formal classification of syndromes by the ILAE.

In general, ESp create the most common early-onset epileptic encephalopathy (EOEE). The outcome is usually poor in 70% of cases [8,9,10]. Prognosis depends on the underlying etiology, which is diverse and includes infections, perinatal events and genetic disorders. Clinical manifestation of West syndrome is: ESp, hypsarrhythmia on EEG and developmental cessation (two out of three criteria are necessary for diagnosis). According to the ILAE, ESp are seizures represented in focal, generalized and unknown onset categories and the distinction may require video-EEG recording [10].

Reviewing the CDG literature, the CDG subtypes reported with ESp /West syndrome are summarized in Table 1.

A detailed description of seizures’ semiology and electroencephalogram abnormalities are scarce in the presentation of the CDG patients with ESe and ESp. Pereira et al. described five patients with ESp and with different types of CDG (ALG1-CDG, ALG6-CDG, ALG11-CDG, CDG-IIx) [15]. The common feature in EEG tracing was slowing of the background activity, lack of hypsarrhythmia, posterior abnormalities (abundant spikes, polyspikes, fast rhythmic burst with lateralization) [15]. Also, the semiology of ESp was different from the classical one, with asymmetry and asynchrony and with associated symptomatology (chewing, facial fear expression, hypersalivation). Clinically ESp were sometimes coupled with myoclonia.

#### 2.1.1. ALG1-CDG

Mutations in the *ALG1* gene were described in 39 patients [31,32,33,34,35]. Morava E et al. in 2012 described refractory epilepsy in 10/14 patients with onset within the first year of life, and recognizable phenotype: microcephaly, developmental delay, strabismus, abnormal blood coagulation tests [36]. To date, the largest group of ALG1-CDG patients were described by Ng et al. [37]. Seizures /epilepsy was noted in 36/38 (95%) [37]. No further clinical details are unfortunately presented.

#### 2.1.2. ALG3-CDG

ALG3-CDG is a very rare CDG subtype with 24 patients described so far. The clinical spectrum comprise mental retardation (22/24), hypotonia (20/24), ESe, microcephaly (19/24), facial dysmorphism, skeletal anomalies and ophthalmological abnormalities [38]. ESe, frequently preceded by fever, were described in 16/24 patients [38]. There is no particular seizures’ type connecting with ALG3-CDG (generalized tonic-clinic, focal, absence, migratory seizures or ESp). Most of the patients presented with burst-suppression pattern or hypsarrhythmia on EEG. Levetiracetam (LEV), vitamin B6, vigabatrin (VGB), clobazam (CLB), phenobarbital (PB) were ineffective [38,39]. Paketci et al. reported successful treatment with ketogenic diet (KD) in twins with ALG3-CDG [39].

#### 2.1.3. ALG11-CDG

Regal et al. [40] described two new ALG11-CDG patients presented with ESe and suppression burst activity on EEG and good response to topiramate (TPM) therapy (previous reports were published by Rind et al. [18] and Thiel et al. [41]), abnormal muscle tone with peripheral spasticity, psychomotor delay, microcephaly. Additional three cases were described in 2017 [15,42]. The first patient observed by Haanpää had been treated because of ESp (extension type) with hypsarrhythmia on EEG (modified hypsarrhythmia with repeated bilateral spikes in temporal and central regions), a second one with severe myoclonic epilepsy of infancy (first seizures were described as startling or freezing episodes then morning generalized seizures) [43]. Nothing is stated about ASM. ESe started in both patients at the age of 3–4 months. According to literature data ESe were present in all 12 patients with ALG11-CDG reported so far [43].

#### 2.1.4. ALG13-CDG

ALG13-CDG, known as an early infantile epileptic encephalopathy-36 (EIEE36), is caused by heterozygous mutation in the *ALG13* gene, located on chromosome Xq23 [12]. Asparagine-linked glycosylation 13 encodes a protein that heterodimerizes with ALG14 to form a functional UDP-GlcNAc glycosyltransferase in the endoplasmic reticulum [12]. Protein asparagine N-glycosylation is thought to be essential for the structure and function of glycoproteins because it catalyzes the second step of protein N-glycosylation. That is why patients present with variable multisystem phenotype.

The Epi4K Consortium and Epilepsy Phenome/Genome Project reported 2 unrelated girls with the onset of seizures at ages 1 and 4 months and showed hypsarrhythmia on EEG [19]. Later on, Michaud et al. described a girl with EIEE36, diagnosed with focal ESp at age 4 months. EEG showed hypsarrhythmia and multifocal discharges [44]. Smith-Packard et al. presented a 7-year-old girl with ESp associated with EEG abnormalities [45]. The seizures remitted until age 5 years when they became more severe and difficult to control. According to Michaud et al., Smith-Packard et al. in about half of ALG13-CDG patients an initial positive response to treatment was observed during ACTH therapy [44,45]. Recurrence of seizures required modification of ASM, introducing TPM, KD. Dimassi et al. reported a 6-year-old girl who developed ESp associated with hypsarrhythmia at age of 2 months [46]. EEG showed slow background activity with multifocal spikes and spike-wave discharges. All above-mentioned girls were diagnosed with psychomotor/intellectual delay. There are also two male ALG13-CDG patients reports with refractory epilepsy with polymorphic seizures [47] and another with c.320A>G variant in ALG13 with ESp and hypsarrhythmia on EEG, treated with VGB, prednisolone, valproic acid (VPA), nitrazepam (NZP), lamotrigine (LTG). In the last patient the best clinical response was due to LEV [48]. Most patients with this mutation, as stated above, were girls showing initially ESp, successively developing myoclonic-tonic spasms, focal seizures, generalized seizures. In 2020, Ng et al. provided information on 29 unreported ALG13-CDG patients, among them, 34 females and 2 males harbor c.320A >G (p.Asn107Ser) variant [49]. Most patients presented with ESp responding to ACTH (87%) or prednisolone therapy (38%) and later benzodiazepines (clonazepam CZP, NZP, CLB) with most commonly used CLB (7–88%) and felbamate (FLB) [49]. Also ketogenic diet gave a sustained antiepileptic effect in 27% of patients. In 87% of patients, hypsarrhythmia was the initial abnormality seen on EEG. Based on literature data Ng et al. concluded that epileptic phenotype in ALG-13 is very homogenous with the mean age of ESp appearance around 6.5 months [49]. Patients’ observation allowed for the suggestion of the first line treatment of ESp in CDG, which is ACTH or prednisolone.

ALG13-CDG clinical spectrum apart from epilepsy covers developmental delay (100%), intellectual disability (92%), hypotonia (85%), coagulation abnormalities and endocrine dysfunction [49].

#### 2.1.5. DOLK-CDG

Mutations in the *DOLK* gene presented with variable symptoms, ranging from nonsyndromic dilated cardiomyopathy to severe multiorgan involvement. Helander et al. described a patient with epilepsy with the onset around 4 months and polymorphic seizures: generalized tonic-clonic seizures, focal seizures, ESp [20]. Due to diagnosed West syndrome pyridoxine, TPM, ACTH were introduced and were partially effective. After modification of treatment, on VGB and CZP therapy the ESe stopped at the age of 6 months [20]. The EEG tracing improved from hypsarrhythmia variant (the irregular slow activity of high amplitude with multifocal epileptic discharges) to normal result.

#### 2.1.6. DPAGT1-CDG

Around 40 patients with DPAGT1-CDG were described so far. Among *DPAGT1* gene mutations, two distinct phenotypes have been highlighted: a systemic disease with encephalopathy or congenital myasthenic syndrome with tubular aggregates in muscle biopsy. 54% of patients were treated because of early-onset epilepsy, four of them were diagnosed with West syndrome [21,50]. 30% of the children died before the age of 12 months. The frequency of seizures, seizure progression, profound hypotonia and psychomotor retardation place DPAGT1 as one of the most severe, lethal type CDG [50].

#### 2.1.7. MPDU1-CDG

*MPDU-1* mutations block the efficient use of Dol-P-Man and Dol-P-Glc in mammalian cells. A first description of this very rare CDG type was given by Schenk et al. [22]. In the description of 7 patients presented so far, severe epilepsy phenotype with apnea, hypertonic seizures in infancy (with good reaction on VPA) and febrile seizures were mentioned. In the EEG tracing multifocal sharp waves, spikes or generalized background slowing dominated [51]. The clinical picture was complemented by psychomotor retardation, hypotonia, facial dysmorphism, eye defects, apnea and skin abnormalities such as ichthyosis.

#### 2.1.8. ST3GAL3-CDG

In 2019, Indellicato et al. described two siblings-one at the age of 7 months, with several episodes of brief psychomotor arrest with ocular deviation [52,53]. EEG showed diffuse epileptiform discharges. A further EEG recorded frontal sharp and spike epileptiform anomalies, both isolated and organized in sequences. In clinical episodes of clonus of the head and upper limbs, EEG showed the organization of the anomalies in a rhythmic and hypersynchronous sequence. VPA and CLB turned out to be effective. His sister, at 8 months of life, experienced similar episodes. Her EEG showed diffuse polymorphic epileptiform abnormalities. VPA and ethosuximide (ETH) were used with clinical improvement. Previous reported ST3GAL3-CDG patients [23] were diagnosed with West syndrome evolving to Lennox-Gastaut syndrome. In 4 members of a consanguineous Palestinian family with early infantile epileptic encephalopathy-15, Edvardson et al. [23] identified a homozygous mutation in the *ST3GAL3* gene and noted that the phenotype was more severe than that observed by Hu et al. [54]. The regiment of drugs was as follows: in 3 patients-VGB, ACTH, VPA, CLB, LEV, two additionally received rufinamide (RFM), the last one without ACTH was also treated with carbamazepine (CBZ) [24]. Of course, the treatment used depended on the dominant semiology of the seizures.

#### 2.1.9. SLC35A2

SLC35A2 belongs to an SLC35A family of nucleotide sugar transporters, which also includes SLC35A1 (CMP sialic acid), SLC35A3 (UDP-N-acetylglucosamine) and two nucleotide sugar carriers with undefined function (SLC35A4 and SLC35A5) [55]. Mutations in the X-linked UDP-galactose transporter gene leads to SLC35A2-CDG resulting in defective galactosylation of glycoconjugates in the Golgi. SLC35A2-CDG may manifest with West syndrome, dysmorphic features and absence of hepatic and coagulation features usually observed in CDG. In a cohort of 30 patients with SLC35A2-CDG, Ng et al. observed major clinical features like developmental delay (100% of patients), ESe (83%), hypotonia (93%), brain abnormalities on neuroimaging (83%; white matter abnormalities-54%, cerebellar atrophy-57%), microcephaly (43%) [55]. Neither the semiology of ESe, EEG or treatment was mentioned. In 2018 Vals et al. described another 15 patients with the same mutation [56]. Epilepsy was present in 80%, in 9 children hypsarrhythmia or findings consistent with unspecified epileptic encephalopathy [56]. No information about the semiology of seizures or antiepileptic therapy were found. 78% of this cohort showed cerebral, cerebellar, pons or brainstem atrophy or delayed myelination. Based on the above information *SLC35A2* gene has been postulated as a candidate gene for epileptic encephalopathy [55,56].

#### 2.1.10. RFT1-CDG

RFT1-CDG is a type of CDG with 11 patients described in the literature. It is another cause of unspecified epilepsy (in some patients with ESp semiology), developmental delay, deafness. Aeby et al. presented a detailed description of two patients with RFT1-CDG [26]. EEG patterns had worsened with age. At the age of two months, the EEG revealed suppression-burst activity (SBA) coupled with clinical numerous tonic seizures [26]. The seizures were transiently controlled with VGB, VPA proved to be ineffective. Another RFT1 child experienced polymorphic seizures on the second day of life: recurrent tonic-clonic seizures with apnea, bradycardia and desaturation [57]. In that case antiepileptic therapy (PB, pyridoxine, phenytoin (PHT), LEV, midazolam, VGB) including corticosteroids were ineffective. At 3 months of age, the EEG worsened to SBA pattern. Because of dominating partial seizures, LEV was re-introduced, later KD and CZP were added because of partial seizures and asymmetric ESp on waking time. The last modification of treatment with TPM and VGB allowed for the cessation of ESe. In RFT1-CDG epilepsy range from severe form [58,59,60] to easy controlled seizures [58,61].

#### 2.1.11. PIGA-CDG

Among 73 individuals with PIGA-CDG reviewed by Bayat et al., myoclonic and/or tonic seizures were present in 38 and focal seizures in 28, ESp were described in 17, six experienced absence seizures, atonic seizures were described in three and migrating focal seizures in two, one suffered from gelastic epilepsy [62]. The febrile-induced seizures, which were followed by unprovoked and poorly controlled ESe, were present in 26; most presented with febrile seizures at onset [62].

Referring to AEM, ESe were intractable in 51 patients among 73 described. In 11 patients, ESe ceased on a combination of different AEM [62]. Additionally, six patients experienced a partial effect from drugs. Among these 51 patients, 21 were placed on a ketogenic diet and eight remained seizure-free, while five patients experienced a significant reduction in seizure frequency [62].

In summary, X-linked PIGA mutations present with ESp with hypsarrhythmia and profound developmental delay. In the severe forms also brain abnormalities (thin corpus callosum and delayed myelination) have been reported [63,64].

#### 2.1.12. PIGW-CDG

Among 8 individuals with PIGW-CDG described to date, clinical data concerning seizures are available for six patients. According to the review by Peron et al., West syndrome was noted in 3, of whom in one individual it evolved to focal-onset seizures, otherwise focal-onset seizures were noted in one of six, two others suffered from atonic seizures [63].

#### 2.1.13. PIGN-CDG

To date, a total of 29 patients with PIGN-CDG and 22 different *PIGN* variants have been reported [64]. Nineteen of them were diagnosed with multiple congenital anomalies-hypotonia-seizures syndrome 1 (MCAHS1), 10 were diagnosed with Fryns syndrome characterize by congenital diaphragmatic hernia (CDH), dysmorphic facial features, pulmonary hypoplasia and other various internal malformations [64]. Fryns syndrome is associated with fatal course, epilepsy in those individuals has not been delineated.

Of 7 patients analyzed recently by Jiao, the first seizures began between 3 months and 1 year of age Focal seizures occurred in five individuals, while other seizure types have also been noted, included atypical absence (3/7), myoclonic seizures (2/7), partial secondarily generalized tonic-clonic seizures (1/7) and generalized tonic-clonic seizures (1/7) [64]. The first seizures occurred in the course of febrile illness in five patients, after which febrile or afebrile seizures developed. In another case, although Ese developed without fever, almost all the subsequent seizures appeared during fever period. In the initial interictal EEG diffuse slow waves with multifocal discharges dominated. The most frequently used antiepileptic drugs were: VPA, TPM, LEV, then oxcarbazepine (OXC) [64].

#### 2.1.14. ST3GAL5

GM3 synthase encoded by *ST3GAL5* sialylates lactosylceramide to GM3, which is the root structure for the most biologically relevant gangliosides. Bowser et al. studied a cohort of 50 patients with ST3GAL5 c.862 C>T (median age 8.1years, range 0.7–30.5years) [65]. The semiology of ESe was as follows: generalized tonic-clonic seizures (48% of patients), generalized non-convulsive (37%), complex partial (20%), tonic spasms (20%), behavioral arrest (16%), ESp (12%), gelastic seizures (4%), atonic (4%), status epilepticus (20%) [65]. In EEG tracing four abnormalities dominated: multifocal spike-wave discharges (97%), slow, high-voltage, disorganized background (2–4Hz, 50–400μV)-75%, absent posterior rhythm (63%), absent sleep-wake architecture (50%) [65].

### 2.2. Ohtahara Syndrome

Both Ohtahara syndrome (OS) and early myoclonic epilepsy (EME) create a group of early-onset encephalopathy (EIEE) with very characteristic EEG of suppression burst activity (SBA) [66,67]. In OS dominating form of seizures are tonic seizures, in EME myoclonic and non-epileptic myoclonic seizures are most frequently seen. Sometimes strict classification into one of these syndromes is difficult, anyway prominent myoclonic seizures at the beginning of epilepsy symptoms point to EME. Pathogenesis also matters. More often OS is connected with congenital brain abnormalities, EME with neurometabolic disorders.

- PIGA-CDG [26]
- PIGQ-CDG, Early infantile epileptic encephalopathy-77 (EIEE77) [66].

A germline mutation in the *PIGA* gene was identified for the first time by Johnston et al. in 2012, by exome sequencing of the X chromosome in a family with multiple congenital anomalies-hypotonia-seizures syndrome-2 [67]. The disease was further delineated by Swoboda K et al., van der Crabben et al. or Kato et al. [27,68,69,70,71]. According to Kato et al., the phenotypic consequences of *PIGA* mutations can be classified into 2 types: severe and less severe, which correlate with the degree of PIGA activity reduction caused by the mutations [27]. Severe forms involved myoclonia and asymmetrical suppression bursts on EEG, multiple anomalies with a dysmorphic face and delayed myelination with restricted diffusion patterns in specific areas [27,72,73]. The less severe forms presented with intellectual disability and treatable seizures (dominant morphology: tonic, clonic and secondarily generalized seizures) without facial dysmorphism. Graovac et al. claimed that the two types of PIGA deficiency share common features like ESp, hypsarrhythmia on EEG, developmental delay [70]. Untill 2020, a total of 76 patients were reported. Eight of them experienced myoclonic seizures usually before the age of 3 months. In EEG most patients showed SBA pattern or hypsarrhythmia. According to Kato et al. and Kim et al. in less severe forms the seizures frequency decreased after two years [27,71]. Kato noticed cessation of seizures on TPM therapy [25].

Martin et al. reported a burst-suppression pattern on EEG and Ohtahara syndrome [66] in PIGQ patients. This was not observed in subsequent patients recognized by Johnstone et al., who described 7 patients with novel pathogenic biallelic *PIGQ* variants [67]. ESe covered with decreasing frequency: tonic seizures, bilateral tonic-clonic (both evolving to status epilepticus), myoclonic seizures, ESp, absence seizures, migrating focal seizures. EEG pattern correlated with Ese’ semiology from burst-suppression, background slowing with interictal sharp waves to hypsarrhythmia and ictal focal spike and slow wave complexes [67].

### 2.3. Early Myoclonic Encephalopathy of Infancy (EMEI)

- PIGA-CDG, Kato [27]
- ALG3-CDG, Fiumara et al. [35]
- ALG6-CDG, Fiumara et al. [35]
- DPM2-CDG, Fiumara et al. [35]
- ALG1-CDG [31,32,33,34,35,74]

Fiumara et al. described CDG patients with EMEI electroclinical features.

In ALG3-CDG patient with cyanotic spells, focal motor and tonic-clonic seizures, PB and VGB were used without seizures’ control. An ALG6-CDG boy experienced sudden episodes of crying followed by apnea and cyanosis, daily tonic seizures with sialorrhea. Treatment with PB, VGB and VPA was unsuccessful [35].

In ALG6-CDG (the most frequent type after PMM2-CDG) polymorphic and difficult to treat seizures develop usually between 5 months and two years: tonic-clonic, partial complex, atonic and myoclonic seizures. PB, VGB VPA, then LEV therapy were found ineffective [35]. Morava et al. collected data on 41 patients with ALG6-CDG, among them 22% developed intractable seizures [14].

In DPM2-CDG, after status epilepticus in neonatal period, daily tonic seizures and limb myoclonia dominated. VPA and LTG have not achieved the seizure control [35].

### 2.4. Epilepsy of Infancy with Migrating Focal Seizures

- ALG3-CDG (Barba et al. 2016) [75]
- ALG1-CDG (Barba et al. 2016) [75]
- RT1-CDG (Barba et al. 2016) [75]

Barba described 4 patients (two patients with *ALG3*, 1 patient with *ALG1*, one patient with *RT1* variant) with migrating seizures between 1 and 4 months, occurring in repeated clusters with prominent clonic or tonic manifestations [75]. In two patients, partial seizures were precipitated by ESp or were accompanied by them. Common EEG features comprise slow background activity, absent sleep stages, multifocal spikes, in three patients additionally spike and waves discharges. All seizures were drug-resistant; the most effective drugs were PB and PHT with >50% reduction of seizures in two patients [75].

## 3. Brain Congenital Abnormalities

Since proper proteins glycosylation is crucial for brain development and glycosylated proteins are essential for CNS functioning [76,77], the frequency and diversity of brain developmental anomalies in CDGs seem understandable. As presented in Table 2, congenital and developmental CNS abnormalities can be divided into cortical malformations, midline brain structures and volume anomalies, myelination disorders and venous sinus thrombosis. Among them, neuronal migration defects (NMD) comprised the largest group [77]. These are traditionally classified based on imaging cortex anomalies into lissencephaly, polymicrogyria, schizencephaly and neuronal heterotopia.

Severe congenital malformations and developmental anomalies, as well as ventriculomegaly/hydrocephalus and myelination disorders, are among common features of several CDG types. The most frequently noted are however corpus callosum anomalies and brain atrophy, usually progressive. Unfortunately, corpus callosum agenesis/hypoplasia (ACC/HCC) seems to have low diagnostics value for CDG, as it isdescribed in about 200 different genetic syndromes, including many CDGs disorders. Hence it is hard to predict any of these diseases based on nonspecific neurologic features with ACC/HCC.

On the contrary, congenital disorders of O-glycosylation are mentioned as a prototype of metabolic disorders associated with neuronal migration defects [77]. In general, severe malformations are most characteristic for muscular dystrophy-dystroglycanopathy (MDDG) syndromes. Among structural brain disorders, pachygyria, polymicrogyria and lissencephaly should always be kept in mind in evaluation of patients suspected of CDG. Some of these malformations may be even characteristics for specific types. Type II cobblestone lissencephaly is known to be mainly associated with O-glycosylation defects [79] and is most frequently described in patients with variants in *POMT1*, *POMT2*, *POMGNT1*, *LARGE* and *FKRP* genes. According to some authors, cobblestone-like brain dysgenesis may be a suggestive feature, example for ATP6V0A2-CDG or brain malformations for DPAGT1-CDG [56]. Rarely, cortical dysplasia or heterotopia are present. The least characteristics are pontocerebellar hypoplasia, described only in PMM2-and B3GALNT2-CDG and venous sinus thrombosis, noted in patients with PMM-and PGM1-CDG.

The mechanisms behind brain malformations are not fully resolved yet. The researchers showed for example, those post-translational modifications of alpha-dystroglycans (α-DG) are essential for normal development of the brain and eyes [80]. The disruption of the proper glycosylation of the alpha DG, which is essential for corticogenesis, is thought to be the reason for the DG neuronal migration defect observed in FKRP-related muscular dystrophies. The importance of functional glycosylation of a-DG in the pathogenesis of is not clearly understood.

Disorders of myelination and progressive brain atrophy are, in turn, somewhat more commonly reported in other types, for example, in glycosylphosphatidylinositol biosynthesis defects (GPIBDs). In most GPI deficiency patients, in the absence of cortical malformations, the high prevalence and severity of early-onset epilepsies are supposed to result from anchored-protein defects/deficiency.

Cerebellar developmental anomalies, as hypoplasia and atrophy, are an important and common feature of various forms of CDG. Most frequently they are described in PMM2-CDG and several types of dystroglycanopathies. Another sign, being characteristic mostly of the latter group of diseases but also seen in SRD5A3-CDG, are cerebellar cysts, which may serve as an important diagnostic clue.

White matter abnormalities are not among the most frequent and typical characteristics of CDGs, they have however been noted in several affected individuals. Feraco et al. have performed MR imaging studies of 5 children (3 boys and 2 girls, aged 12 days to 2 years at presentation) with molecularly confirmed PMM2-CDG [82]. In 4 of them, the ce-rebellar cortex and subcortical white matter were hyperintense on T2-weighted and FLAIR images [82]. In a 5-year-old girl, diagnosed with B3GALNT2-CDG, increased T2 signal from supratentorial and infratentorial white matter at the age of 3.5 years were noted [83]. In 2019, the most common brain MRI abnormalities described in a group of 15 patients (11 females and 4 males) with SLC35A2-CDG were cerebral atrophy with delayed myelination and multifocal inhomogeneous abnormal patchy white matter hyperintensities, which seemed to be nonprogressive [56]. Leukodystrophy has been recently reported by Larsen and colleagues [84]. Nevertheless, it was questioned by others [85].

## 4. Microcephaly

Microcephaly as a feature of CDG was already mentioned above, in individuals diagnosed with pathogenic variants in *DPM2* [14], *ALG11* [41], *SLC35A2* [55], *ALG1* [36] and *ALG3* [38] genes. It is frequently observed in CDGs, which manifests usually at birth. In our clinical experience it has never been seen as a postnatal progressive finding.

Except mentioned above diseases, other rare CDG types, as COG6-CDG [86,87], oligosaccharyltransferase complex-congenital disorders of glycosylation [88] and COG2-CDG [89] have been recently clinically expanded to include microcephaly. In 2019, Mulkey et al. present a prenatal case with arrest of brain development, consistent with fetal brain disruption sequence, previously unreported feature of congenital microcephaly in ALG11-CDG [90]. The fetal biometry at 20-week of gestation gave normal result. At the following screening, at 36-week, microcephaly was noted. Fetal magnetic resonance imaging showed severe cortical thinning with a simplified gyral pattern (as for gestational age), agenesis of the corpus callosum and ventriculomegaly. As characteristic of fetal brain disruption sequence, the skull had a posterior shelf at the level of the lambdoid suture. Postnatal brain neuromagnetic revealed no brain growth from the fetal to postnatal study.

Péanne et al. summarized that microcephaly could be a feature of ALG9-CDG, Gillessen-Kaesbach-Nishimura syndrome, ALG12-CDG, ATPVO2-CDG, Wrinkly skin syndrome, COG1-CDG, COG4-CDG, COG7-CDG, DPAGT1-CDG, DPM1-CDG, MGAT2-CDG, NUS1-CDG, PGAP1-CDG, PGAP2-CDG, PGAP3-CDG, PIGY-CDG, RFT1-CDG, SLC35A1-CDG, SLC35A3-CDG, SLC35C1-CDG, ST3GAL5-CDG, STT3A-CDG, STT3B-CDG, TMEM5-CDG [4].

## 5. Developmental Delay (DD) and Intellectual Disability (DD/ID)

DD and ID are frequently observed characteristics of numerous CDGs, that was mentioned in earlier sections (i.e., PMM2-CDG, PIGA-CDG, ALG13-CDG, LARGE-CDG, DOLK-CDG). Based on our experience, individuals with PMM2-CDG improve with development support and have mild/moderate disability. We do not see any regression or lack of progress in development.

DD/ID are not however constant features and milder spectrums have also been reported as in other types of glycosylation disorders, as in those listed. For example, a 10-year-old boy presented with the multi-systemic manifestation involving certain distinct clinical features of CDG but mild ID was diagnosed with PGM1-CDG [91]. Normal cognitive development has been also reported in single individuals suffering from ALG13-CDG [92] and six patients (from three families) with mild PMM2-CDG [93]. In 5/6 children from the families the Wechsler Intelligence Scale for Children (WISC) showed normal cognitive development with full scale IQ scores ranging from borderline to average. A boy described by Gadomski et al. [92] had ESe, delayed motor and speech development but cognition was stated as normal. Most recently, a novel homozygous *ALG12* variant (c.77T>A, p.Val26Asp) was identified in a 25-year-old women without ID. In all these patients mild (or lack of) neurological impairment is an uncommon feature that extends the phenotype CDGs with multiorgan involvement [94].

## 6. Ataxia

Cerebellar developmental anomalies, as hypoplasia and atrophy, are an important and common feature of various forms of CDG (Table 2). Most frequently they are described in PMM2-CDG and several types of congenital muscular dystrophies due to dystroglycanopathy. Another sign, being characteristic mostly of the latter group of diseases but also seen in SRD5A3-CDG, are cerebellar cysts, which may serve as an important diagnostic clue (Table 3).

Ataxia, apart from being a feature of more commonly diagnosed CDGs (e.g., *ALG6*, *COG8*, *DPM1*, *PMM2)*, may be also a feature of *COG4*, *COG5*, *GMPPB*, *MPDU1*, *NANS*, *PGM3*, *SLC35A1*, *TRAPPC11* /Muscular dystrophy, limb-girdle, type 2S/. The question regarding the etiology of ataxia in the course of CDG is still open. Some authors postulated recently, the role of CaV2.1 channel [95]. The hypoglycosylation of its αbδ subunit, by the gain-of-function effect, may promote the voltage-dependent opening of the CaV2.1 channel, leading to ataxia. A similar mechanism is postulated to result in stroke-like, observed in some CDG individuals (discussed in the below section). Since N-glycosylation of CaV2.1 may participate in cerebellar syndrome in PMM2-CDG, acetazolamide therapy was suggested [96]. AZATAX (AZATAX: Acetazolamide Safety and Efficacy in Cerebellar Syndrome in PMM2 Congenital Disorder of Glycosylation, PMM2-CDG) was designed to establish whether acetazolamide is safe and improves cerebellar syndrome in PMM2-CDG. This first clinical trial of PMM2-CDG showed evidence that acetazolamide is well tolerated and effective for the motor cerebellar syndrome. Moreover, its potential in preventing stroke like episodes (SLEs) and its long-term effects on kidney function was proposed to be addressed in future studies.

## 7. Neuromuscular Presentation

A variety of neuromuscular symptoms in CDG have been reported. Several CDG types combine myasthenic features with other symptoms of CNS involvement. In dystroglycanopathies defects are located in the O-mannosylation of the sarcolemmal protein alpha-dystroglycan. Mutations in DPAGT1 can show myasthenic syndrome with limb-girdle pattern [97]. *ALG2* mutations may cause varying onset of myasthenic syndrome with myopathic features. In ALG14-CDG Cossins et al. identified childhood-onset isolated myasthenic syndrome with limb-girdle pattern [98]. Later on, Schorling et al. described five ALG14-CDG patients with the lethal phenotype (from 3 families) and dominant clinical features were myasthenic and myopathic features coupled with cerebral atrophy and refractory epilepsy [99]. MRI showed delayed myelination with frontoparietal atrophy in 4 patients and in one white matter loss and ventriculomegaly.

DPM1-CDG, DPM2-CDG, DPM3-CDG are connected with hypoglycosylation of alphadystroglycan coupled with elevated creatine kinase level and muscle dystrophy. Congenital muscular dystrophy is also linked with following genes: *POMT1*, *POMT2*, *POMGNT1*, *FKTN*, *FKRP*, *LARGE*, *ISPD*, *GTDC2*, *B3GNT1*, *GMPPB*, *SGK196*, *TMEM5* [4]. This group of dystroglycanopathies may range in phenotype from Walker–Warburg syndrome, a severe, congenital form with brain involvement to milder forms of limb girdle dystrophies.

## 8. Spasticity

Most often congenital disorder of glycosylation are associated with hypotonia. ST3GAL5-CDG named “salt and pepper” syndrome apart from spasticity is characterized by severe intellectual disability, dysmorphic features, seizures, scoliosis, choreoathetosis and altered dermal pigmentation [65]. Mutations in *B4GALNT1* gene encoding GM2/GD2/GA2 synthase have been reported in association with hereditary spastic paraplegia HSP26 [100]. Péanne et al. mentioned about the possibility of spastic quadriplegia in COG-CDG and peripheral hypertonia in PIGP-CDG [4].

## 9. Peripheral Neuropathy

In general, peripheral neuropathies in CDG, are frequently labelled as Charcot-Marie-Tooth disease-like picture. Peripheral neuropathy is present in 53% of PMM2-CDG patients [7,101]. Neurophysiology studies in PMM2-CDG described by Freeze et al. showed slowing of motor-nerve conduction with sparing of the sensory-nerve conduction [74,102]. In general, peripheral neuropathies in CDG are frequently labelled as Charcot-Marie-Tooth disease. In NGLY1-CDDG the most common presentation is global developmental delay, hyperkinetic movement disorder, peripheral neuropathy, alacrimia/hypolacrimia [103]. Based on experimental studies impaired axonal guidance and loss of the myelinated nerve fibers are suggested to be responsible for peripheral neuropathy in this congenital disorder of deglycosylation.

## 10. Movement Disorders

Movement disorders in children are very heterogeneous group with the respect to motor semiology and underlying pathology. They are frequently classified into: hyperkinetic (stereotypies, tics, tremor, chorea, dystonia) and hypokinetic (parkinsonian phenotype) forms. Based on first detailed observation of eight CDG patients (PMM2-CDG, ALG6-CDG, ALG8-CDG, COG5-CDG) with age ranging from 2 to 28 years, Mostile et al. documented abnormal involuntary movements starting from infancy period [104]. The detected movements were exacerbated by emotional stress, fatigue. The authors suggested that hyperkinetic movement disorders in CDG may be more frequent than previously thought due to lack of earlier investigation [104]. Hyperkinetic movement disorders in CDG are summarized in Table 4.

## 11. Stroke-Like Episodes (SLEs)

SLEs are most frequent in PMM2-CDG (20–55% of PMM2-CDG patients). In neuroimaging the involved areas do not correspond to classic vascular distribution, involving predominantly the temporal, parietal and occipital lobes and sometimes also subcortical white matter [95,111]. It is not explained by the coagulation and perfusion abnormalities seen in CDG. Izquierdo-Serra et al. in 2018 presented 43 PMM2-CDG patients with 9 SLE events [95]. They suggested two triggering factors: head trauma and viral infection. In 8 patients on brain MRI during SLE, no acute injury was observed, 1 patient revealed diffuse cortical edema in the parieto-occipital region of one hemisphere. The duration of a single episode (confusional state, mono-or hemiparesis, sometimes ESe, dysphasia, dysphagia, conjugate eye deviation, blindness) may be 1h to several months [95]. Complete recovery (within one hour to several months) is observed in the majority of the patients but exceptionally residual motor abnormalities may persist. Usually, good hydration and ASM epileptic treatment are sufficient. Based on observation of 96 patients Schiff et al. concluded that SLE appeared in 12 patients (12,5%) as a localized zone with restricted diffusion (cytotoxic oedema) [7].

SLE may also complicate the course of other neurological diseases such as channelopathies related to Familial Hemiplegic Migraine (FHM), a paroxysmal neurological disease caused by mutations in *CACNA1A* (encoding the neuronal pore-forming CaV2.1 channel α1A subunit), *ATP1A2* (encoding the Na^+^, K^+^-ATPase pump α2 subunit) or SCN1A (encoding the neuronal NaV1.1 channel α1 subunit). The mechanisms behind SLE in CDG are still unknown. One of the current hypotheses assumed the abnormal Ca_V_2.1 function-(encoded by the *CACNA1A* gene), resulting from aberrant *N*-glycosylation, as a potential novel pathomechanism of SLE [95]. A similar mechanism is postulated as a cause in ataxia, observed in PMM2-CDG individuals (discussed in the above section).

It is worth to mention, that the term SLE was coined for mtDNA point mutations associated with MELAS (mitochondrial myopathy, encephalopathy, lactic acidosis, stroke-like episodes). The diagnosis is definite with at least two category A criteria (headaches with vomiting, seizures, hemiplegia, cortical blindness and acute focal lesions in neuroimaging) and two category B criteria (high plasma or cerebrospinal fluid (CSF) lactate, mitochondrial abnormalities in muscle biopsy and a MELAS-related gene mutation) [112]. The pathomechanism of SLE in MELAS is connected with angiopathy due to mitochondrial proliferation in smooth muscle and endothelial cells of small blood vessels and impaired blood perfusion in cerebral microvasculature [112].

## 12. Autistic Spectrum Disorder

Abnormal social interactions, limited interests and stereotypic, repetitive behaviors which all are characteristics of autism spectrum disorders (ASDs) [112] are not typical feature of CDG. A potential link between ASDs and congenital disorders of glycosylation was however pointed in patients with CDGs [74]. Now, some CDG types have been recognized as presenting with ASD [76], with most frequent presented in Table 5.

As noted above, among CDGs being associated with autistic spectrum disorder, mainly those affected downstream steps in N-and O-glycan biosynthesis were described [76]. Thus, likely alterations in their structure instead of loss of chains are observed. Other mechanisms may also be causative as far as isolated ASD is discussed, like 3-O-sulfation of heparan sulfate chains, degradation of heparan sulfate, sulfation of terminal galactose residues on N-and O-glycans or branching of type II polylactosamine units found on N-glycans.

## 13. Retinitis Pigmentosa

Retinitis pigmentosa is not a constant feature of CDG (Table 6). The disease should, however, be included in the differential diagnosis, especially in the evaluation of patients with seizures, ataxia, areflexia, developmental delay (ALG6-CDG) or be evaluated in individuals diagnosed with PMM2-CDG [113].

## 14. Conclusions

Based on experimental studies, disordered glycosylation process, despite the impact on glycoprotein and glycolipid metabolism, may also secondary influence on other cellular pathways. Protein glycosylation is extremely important for cell interaction, membrane structure, immunity, cell migration during fetal development. The postulated impact of glycosylation deficiency on amino amino acids, phospholipids, sphingolipids, complex fatty acid biosynthesis and remodeling is now verified in further metabolomic analyses [119,120,121,122]. Due to possibility of overlapping phenotypes the recognition of CDG may be difficult especially in young age, in early stages of the disease.

Clinical presentation and course of congenital disorders of glycosylation are incredibly variable, from the global DD with epilepsy to subclinical coagulopathy [119,120,121]. However, some recognizable patterns have emerged and some manifestations are widely known, for example, inverted nipples and abnormal distribution of adipose tissue over the buttocks or suprapubic region and cerebellar hypoplasia in PMM2-CDG, achalasia and alacrimia without adrenal insufficiency in GMPPA-CDG, increased alkaline phosphate (ALP) in selected Inherited GPI Deficiency disorders (IGDs) (named after that Hyperphosphatasia with Mental Retardation Syndrome HMRS) and [122], as well as others in the latter group, that is, vertical nystagmus noted in PIGN-CDG patients [123], aplastic/hypoplastic nails or CHIME syndrome [123].

CDG are frequently divided into multisystemic (affecting heart, endocrine and immune system, gastrointestinal tract, skeleton, coagulation parameters) or primary neurologic presentation though defining the specific phenotype is additionally hindered by the fact that for many CDG subtypes only very few patients are known [90]. In parallel, because of the additional diversity of glycosylation pathways involved, biomarkers are limited to subgroups of CDG types. Several CDG types with defects in the N-glycosylation pathway are known because of normal transferrin profile: TUSC3-CDG, ALG13-CDG, ALG14-CDG, GCS1-CDG, SLC35A3-CDG, SLC35C1-CDG, GNE, PGM3, GFPT1 [119,120,121,122]. Though recently, an abnormal profile has been reported in 2 female patients with ALG13-CDG [124].

Given the clinical heterogeneity, the highest diagnostic yield for CDG is genetic testing using next-generation based methodology, especially whole-exome sequencing (WES) or clinical exome sequencing (CES). It allows for diagnosis, discovery novel, rare CDG types and identification of *de novo* mutations. The genetic diagnostic process must be however supported by detailed clinical analysis, including physical examination (presence/absence of facial dysmorphic features, minor congenital anomalies, like brachydactyly, hypoplastic fingernails, inverted nipples), family and obstetric history, assessment of major congenital malformation (especially within central nervous system), detailed ocular and neurological evaluation (concentrated on medical history of seizures) and laboratory findings (ALP, IEF, liver enzymes, coagulation disorder) or flow cytometry (e.g., in novel variants consistent with a diagnosis of IGD) [119,120,121]. Specialised biochemical tests including measuring enzyme activities, or performing in depth analysis of the glycosylation profile can be used as confirmtory tests of the genetic diagnosis. 

Apart from above mentioned CDG types connected with special forms of epileptic encephalopathies also in other CDG types epilepsy may be difficult to treat for example: ALG2-CDG, ALG8-CDG, ALG9-CDG, ALG12-CDG, DPM1-CDG, MPDU1-CDG, DPAGT1-CDG [1,2,3,4,5,6,119,120,121]. There is a lack of long-term follow-up of CDG patients with epilepsy to accurately determine its course. Antiepileptic treatment in CDG does not deviate from the treatment standards depending on the semiology of the ESe or diagnosed epileptic syndrome. The question is whether the division and ranking of types of CDG, types of mutations for a given epileptic syndrome is justified. It is assumed that severe epileptic encephalopathies evolve from Ohtahara syndrome, through West syndrome to Lennox-Gastaut syndrome. The morphology of the seizures changed with the child’s age. The tendency towards neurometabolic epilepsies has been reported to decrease in some affected individuals with age. Does this happen similarly with epilepsy in CDG syndrome?

From a neurological perspective, it is recommended that CDG should be taken into consideration in the diagnosis of a patient presenting with an unexplained multisystemic involvement, encompassing epilepsy of early onset and/or febrile seizures, DD, brain malformations (also cobblestone-like brain dysgenesis) and/or dysmorphic features.

## Figures and Tables

**Table 1:** Congenital disorders of glycosylation (CDG) types connected with Epileptic Spasms (ESp)/West Syndrome (the names of the diseases are given following the Online Mendelian Inheritance in Man^®^, OMIM nomenclature).

Disorder, #OMIM	Gene, Early Infantile Epileptic Encephalopathy (EIEE)	Enzyme	First Description of West Syndrome/Epileptic Spasms
N-linked pathway or multiple			
ALG1-CDG, #605907	*ALG1*	β1,4 mannosyltransferase	de Koning et al., 1998 [16]
ALG3-CDG, #608750	*ALG3*	α1,3 mannosyltranferase	Kranz et al., 2007 [17]
ALG11-CDG, #613661	*ALG11*	asparagine-linked glycosylation protein 11	Rind et al., 2010 [18]
ALG13-CDG, #300884	*ALG13*,EIEE-36	GlcNAc transferase	Allen et al., 2013 [19]
DOLK—CDG, #610768	*DOLK*	Dolichol kinase	Helander et al., 2013 [20]
DPAGT1-CDG, #608093	*DPAGT1*	UDP-N-acetylglucosamine—dolichyl-phosphate N-acetylglucosaminephosphotransferase	Wu et al., 2003 [21]
MPDU1-CDG, # 609180	*MPDU1*	Mannose-P-dolichol synthase	Schenk et al., 2001 [22]
ST3GAL3-CDG, #615006	*ST3GAL3*, EIEE-15	β-galactoside α-2,3-sialyltransferase 3	Edvardson et al., 2013b [23,24]
SLC35A2-CDG, #300896	*SLC35A2*,EIEE-22	UDP-GlcNAc transporter	Kodera et al., 2013 [25]
RFT1-CDG, #612015	*RFT1*	Putative flippase involved in Man5GlcNAc2-PP-Dol transfer	Aeby et al., 2016 [26]
GPI anchor synthesis			
Multiple congenital anomalies-hypotonia-seizures syndrome 2, # 300868	*PIGA*	Phosphatidylinositol N-acetylglucosaminyltransferase subunit A	Kato et al., 2014 [27]
Hyperphosphatasia intellectual disability syndrome 1, #239300	*PIGV*	Mannosyltransferase	Chiyonobu et al., 2014 [28]
Multiple congenital anomalies-hypotonia-seizures syndrome 1, # 614080	*PIGN*	GPI ethanolamine phosphate transferase	Maydan et al., 2011 [29]
Others			
Salt and pepper developmental regression syndrome, #609056	*ST3GAL5*	sialyltransferase	Simpson et al., 2004 [30]

**Table 2:** Brain malformations, anomalies and cerebral complications characteristic for specific CDG (the names of the diseases are given following the Online Mendelian Inheritance in Man^®^, OMIM nomenclature [4,5,76,77,78,79,80,81].

Cortical Malformations				Midline Brain Structures Anomalies		Brain Volume Anomalies		Myelinization Disorders	Venous Sinus Thrombosis
Pachygyria	Polymicrogyria	Lissencephaly	Cortical Dysplasia, Heterotopia	Corpus Callosum (CC) Anomalies	Pontocerebellar Hypoplasia	Nrain Atrophy	Ventriculomegaly and Hydrocephaly		
*ATP6V0A2*/Cutis laxa, autosomal recessive, type IIA *FKRP*/Muscular dystrophy-dystroglycanopathy (congenital with brain and eye anomalies), type A, 5 *FKTN*/Muscular dystrophy-dystroglycanopathy (congenital with brain and eye anomalies), type A, 4 *LARGE*/Muscular dystrophy-dystroglycanopathy (congenital with intellectual disability), type B, 6 *POMGNT1*/Muscular dystrophy-dystroglycanopathy (congenital with brain and eye anomalies), type A, 3 *POMT1*/Muscular dystrophy-dystroglycanopathy (congenital with brain and eye anomalies), type A, 1 *POMT2*/Muscular dystrophy-dystroglycanopathy (congenital with brain and eye anomalies), type A, 2 [especially frontoparietal]*TMEM5*/Muscular dystrophy-dystroglycanopathy (congenital with brain and eye anomalies), type A, 10	*B3GALNT2*/Muscular dystrophy-dystroglycanopathy (congenital with brain and eye anomalies), type A, 11 *POMGNT1*/Muscular dystrophy-dystroglycanopathy (congenital with brain and eye anomalies), type A, 3 *POMT1*/Muscular dystrophy-dystroglycanopathy (congenital with brain and eye anomalies), type A, 1 *POMT2*/Muscular dystrophy-dystroglycanopathy (congenital with brain and eye anomalies), type A, 2	*B3GALNT2*/Muscular dystrophy-dystroglycanopathy (congenital with brain and eye anomalies), type A, 11 (cobblestone) *FKRP*/Muscular dystrophy-dystroglycanopathy (congenital with brain and eye anomalies), type A, 5 (cobblestone) *FKTN*/Muscular dystrophy-dystroglycanopathy (congenital with brain and eye anomalies), type A, 4 ISPD/Muscular dystrophy-dystroglycanopathy (congenital with brain and eye anomalies), type A, 7 (cobblestone) *POMGNT1*/Muscular dystrophy-dystroglycanopathy (congenital with brain and eye anomalies), type A, 3 *POMT1*/Muscular dystrophy-dystroglycanopathy (congenital with brain and eye anomalies), type A, 1 (cobblestone) *POMT2*/Muscular dystrophy-dystroglycanopathy (congenital with brain and eye anomalies), type A, 2 (cobblestone) *TMEM5*/Muscular dystrophy-dystroglycanopathy (congenital with brain and eye anomalies), type A, 10 (cobblestone)	*ISPD*/Muscular dystrophy-dystroglycanopathy (congenital with brain and eye anomalies), type A, 7 (heterotopia) *POMGNT1*/Muscular dystrophy-dystroglycanopathy (congenital with brain and eye anomalies), type A, 3 (dysplasia) *POMT2*/Muscular dystrophy-dystroglycanopathy (congenital with brain and eye anomalies), type A, 2 (heterotopia)	*ALG3* *ALG6* *COG2* (thin CC) *COG4* (thin CC) *FKRP*/Muscular dystrophy-dystroglycanopathy (congenital with brain and eye anomalies), type A, 5 *FKTN*/Muscular dystrophy-dystroglycanopathy (congenital with brain and eye anomalies), type A, 4 *ISPD*/Muscular dystrophy-dystroglycanopathy (congenital with brain and eye anomalies), type A, 7 [atrophy] *MOGS* [small] *NANS*/Spondyloepimetaphyseal dysplasia, Camera-Genevieve type *PGAP1* *PIGA*/Multiple congenital anomalies-hypotonia-seizures syndrome 2 [thin] *PIGG* [thin] *PIGP* [thin] *POMGNT1*/Muscular dystrophy-dystroglycanopathy (congenital with brain and eye anomalies), type A, 3 *POMT1*/Muscular dystrophy-dystroglycanopathy (congenital with brain and eye anomalies), type A, 1 *POMT2*/Muscular dystrophy-dystroglycanopathy (congenital with brain and eye anomalies), type A, 2 (aplasia)andMuscular dystrophy-dystroglycanopathy (congenital with mental retardation), type B, 2 *SLC35A2* (thin CC) *SSR4* (thin CC) *VPS13B*/Cohen syndrome	*B3GALNT2*/Muscular dystrophy-dystroglycanopathy (congenital with brain and eye anomalies), type A, 11 *PMM2*	*ALG1* *ALG9* *B4GALNT1*/Spastic paraplegia 26, autosomal recessive(cortical) *B3GALTL*/Peters-plus syndrome *COG1* (cotrex) *COG2* *COG4* *COG5* (cerebellum and cotrex) *COG7* *COG8* *DPAGT1* *DPM1* *FKTN*/Muscular dystrophy-dystroglycanopathy (congenital with brain and eye anomalies), type A, 4 (cortical) *MOGS* *NANS*/Spondyloepimetaphyseal dysplasia, Camera-Genevieve type *NUS1* (cortical) *PGAP2*/Hyperphosphatasia with mental retardation syndrome 3 *PIGG* *PIGN*/Multiple congenital anomalies-hypotonia-seizures syndrome 1 *PIGT*/Multiple congenital anomalies-hypotonia-seizures syndrome 3 *POMT2*/Muscular dystrophy-dystroglycanopathy (congenital with mental retardation), type B, 2 *SLC35A2* *SLC35C1* *SLC39A8* *SRD5A3* *ST3GAL5*/Salt and pepper developmental regression syndrome (cortical) *STT3A* *STT3B* *TMEM5*/Muscular dystrophy-dystroglycanopathy (congenital with brain and eye anomalies), type A, 10 *TRAPPC11*/Muscular dystrophy, limb-girdle, type 2S	*ALG12* (widening of the lateral ventricles) *ALG13* hydrocephalus *B3GALNT2*/Muscular dystrophy-dystroglycanopathy (congenital with brain and eye anomalies), type A, 11(hydrocephalus)? *FKRP*/Muscular dystrophy-dystroglycanopathy (congenital with brain and eye anomalies), type A, 5 (both) *FKTN*/Muscular dystrophy-dystroglycanopathy (congenital with brain and eye anomalies), type A, 4 (hydrocephalus) *ISPD*/Muscular dystrophy-dystroglycanopathy (congenital with brain and eye anomalies), type A, 7 (both) *NANS*/Spondyloepimetaphyseal dysplasia, Camera-Genevieve type (hydrocephalus) *POMGNT1*/Muscular dystrophy-dystroglycanopathy (congenital with brain and eye anomalies), type A, 3 (both) *POMT2*/Muscular dystrophy-dystroglycanopathy (congenital with brain and eye anomalies), type A, 2 (hydrocephalus)	*ALG2* (delayed) *ALG9* (delayed) *ALG13*/Epileptic encephalopathy, early infantile, 36 *DDOST* *DPM1* *PGAP1* (delayed) *PGM3*/Immunodeficiency 23 *PIGA*/Multiple congenital anomalies-hypotonia-seizures syndrome 2 (delayed) *SLC35A2* (delayed)	*PGM1* *PMM2*

**Table 3:** Most frequent cerebellar anomalies and ataxia in specific CDG types (the names of the diseases are given following the Online Mendelian Inheritance in Man^®^, OMIM nomenclature) [4,5,78,81].

Cerebellar Atrophy	Cerebellar Hypoplasia	Cerebellar Cysts
*ALG1* *ALG3* *ALG6* *ALG8* *ALG9* *COG8* *DPM1* *FKRP*/Muscular dystrophy-dystroglycanopathy (congenital with brain and eye anomalies), type A, 5 *FKTN*/Muscular dystrophy-dystroglycanopathy (congenital with brain and eye anomalies), type A, 4 *PIGN*/Multiple congenital anomalies-hypotonia-seizures syndrome 1 *PMM2* *SLC35A2* *SLC39A8* *SRD5A3* *STT3A* *STT3B* *TRAPPC11*/Muscular dystrophy, limb-girdle, type 2S	*ALG1* *ALG3* *DPM2* *GMPPB*/Muscular dystrophy-dystroglycanopathy (congenital with brain and eye anomalies), type A, 14 and Muscular dystrophy-dystroglycanopathy (congenital with mental retardation), type B, 14 *ISPD*/Muscular dystrophy-dystroglycanopathy (congenital with brain and eye anomalies), type A, 7 *PGAP1* *PIGA* *PIGG* *PIGT*/Multiple congenital anomalies-hypotonia-seizures syndrome 3 *PMM2* *POMGNT1*/Muscular dystrophy-dystroglycanopathy (congenital with brain and eye anomalies), type A, 3 and Muscular dystrophy-dystroglycanopathy (congenital with mental retardation), type B, 3 *POMT1* /Muscular dystrophy-dystroglycanopathy (congenital with brain and eye anomalies), type A, 1 and Muscular dystrophy-dystroglycanopathy (congenital with mental retardation), type B, 1 *POMT2*/ Muscular dystrophy-dystroglycanopathy (congenital with brain and eye anomalies), type A, 2 and Muscular dystrophy-dystroglycanopathy (congenital with mental retardation), type B, 2 *VPS13B*/Cohen syndrome	*B3GALNT2* *FKRP*/ Muscular dystrophy-dystroglycanopathy (congenital with brain and eye anomalies), type A, 5 and Muscular dystrophy-dystroglycanopathy (congenital with or without mental retardation), type B, 5 *FKTN*/Muscular dystrophy-dystroglycanopathy (congenital with brain and eye anomalies), type A, 4 *POMGNT1*/Muscular dystrophy-dystroglycanopathy (congenital with brain and eye anomalies), type A, 3 and Muscular dystrophy-dystroglycanopathy (congenital with mental retardation), type B, 3 *POMT1*/Muscular dystrophy-dystroglycanopathy (congenital with brain and eye anomalies), type A, 1 *POMT2*/Muscular dystrophy-dystroglycanopathy (congenital with brain and eye anomalies), type A, 2 *SRD5A3*

**Table 4:** Hyperkinetic movement disorders in CDG (the names of the diseases are given following the Online Mendelian Inheritance in Man^®^, OMIM nomenclature) [2,3,4,7,35,74,102,103,104,105,106,107,108,109,110].

Disorder, #OMIM	Chorea	Athetosis	Dystonia	Stereotypies	Tremor
N-linked pathway					
PMM2, #212065	+	+	+	+	+
ALG3-CDG, #601110			+		
ALG6-CDG, #603147	+	+	+	+	+
ALG8-CDG, #608104	+				
ALG13-CDG, #300884	+				
MGAT2-CDG, #212066				+	
DPAGT1, #608093					+
DDOST-CDG, #614507					+
COG5-CDG, #613612	+			+	
MOGS-CDG, #606056			+		
SRD5A3-CDG, # 612379				+	
N-and O-linked pathways					
TRAPPC11-CDG, # 615356 (Muscular dystrophy, limb-girdle, autosomal recessive 18)	+				+
GPI anchor synthesis					
PGAP1-CDG, # 615802				+	
PIGN-CDG, #614080	+				+
PIGV-CDG, #239300		+	+		
Others					
ST3GAL5, #609056	+	+			+
B4GALNT1, #609195			+		
Disorder of deglycosylation					
NGLY1-CDDG, #615273	+	+	+		+

+ present.

**Table 5:** Autistic spectrum disorder as a feature of CDG [5,14,20,23,76].

Gene	Clinical Characteristics
*ALG6*	autistic features (abnormal communication, difficulties with social interaction, recurrent episodes of repetitive behavior) in 14/41 patients with a diagnosis of autism in five
*DOLK*	intellectual disability and an autism spectrum disorder in one (female) from affected siblings
*SLC35A3*	Mutation in this genewas identified in one large kindred displaying eight individuals with epilepsy, arthrogryposis, developmental delay and autism spectrum disorders [23].

**Table 6:** Retinitis pigmentosa as a feature of genes, causing CDG [5,113,114,115,116,117,118].

Gene/Disease	Clinical Characteristics
*ALG6*	The second largest subtype of CDG, characterized by psychomotor retardation with delayed walking and speech, hypotonia, ESe and sometimes protein-losing enteropathy. Atrophic retinal pigmentation, decreased retinal vascularization were also reported.
*DHDDS*/Retinitis pigmentosa 59	First described in patients with a confirmed diagnosis of CDG in 2016. All previously known individuals with mutations in the *DHDDS* gene presented with a mild clinical picture restricted to retinitis pigmentosa [114].
*PMM2*	The data by Andréasson et al. suggested that patients with CDG have a progressive tapetoretinal degenerative disorder of the retinitis pigmentosa type with defined alterations in the ERG [117].
*POMGNT1*/Retinitis pigmentosa 76	In 2016 Wang et al. presented evidence of the involvement of O-mannosyl glycosylation in isolated retinitis pigmentosa (shows an instance of POMGNT1 mutation that does not involve muscular dystrophy) [118]. The gene, which encodes a glycosyltransferase in O-mannosyl glycosylation, was before found to be responsible for a group of congenital muscular dystrophies called dystroglycanopathies.
